# Update on aneurysmal bone cyst: pathophysiology, histology, imaging and treatment

**DOI:** 10.1007/s00247-022-05396-6

**Published:** 2022-08-09

**Authors:** Ricardo Restrepo, David Zahrah, Liset Pelaez, H. Thomas Temple, James W. Murakami

**Affiliations:** 1 Department of Radiology, Nicklaus Children’s Hospital, Miami, FL USA; 2 School of Arts and Sciences, Washington University, St. Louis, MO USA; 3 Department of Pathology, Nicklaus Children’s Hospital, Miami, FL USA; 4 Department of Orthopedic Surgery, Miller School of Medicine, University of Miami, Miami, FL USA; 5 Department of Radiology, Nationwide Children’s Hospital, 700 Children’s Drive, Columbus, OH 43205 USA

**Keywords:** Aneurysmal bone cyst, Bone, Children, Computed tomography, Magnetic resonance imaging, Radiography, Sclerotherapy

## Abstract

Aneurysmal bone cyst (ABC) is a benign but locally aggressive lesion that predominantly affects children and young adults. ABC, which accounts for approximately 70% of the cases, is now recognized to be a true neoplasm, whereas ABC-like changes associated to other bone neoplasms (also referred in the literature as secondary ABC) accounts for the remaining 30%. The solid variant of ABC is also considered a true neoplasm but is rare. ABC can involve any bone in the body, and although it has a metaphyseal preference, it can involve any part of a bone and soft tissues. As with any bone tumor, the initial evaluation of ABCs should be done with radiographs followed by magnetic resonance imaging or less frequently computed tomography for further characterization. The imaging appearance of ABC is variable; however, a lytic and expansile lesion with fluid-fluid levels is the most common presentation. The main differential diagnosis of an ABC in the pediatric population is unicameral bone cyst (UBC) and telangiectatic osteosarcoma, therefore a biopsy is recommended before treatment. The therapeutic options of ABC range from curettage with or without adjuncts such as phenol, liquid nitrogen, argon laser and bone grafting or bone substitutes to more recently employed alternatives such as image-guided sclerotherapy with various sclerosing agents and monoclonal antibodies (e.g., Denosumab).

## Introduction

Aneurysmal bone cyst (ABC) is a descriptive term in which the word “aneurysmal” refers to the marked expansion and the word “cyst” refers to “fluid-filled cavities.” However, the lesion that is now considered a true neoplasm is neither an aneurysm nor a cyst. ABC accounts for approximately 1% of all bone tumors with a reported incidence of 0.14 per 100,000 individuals and a prevalence of 0.32 cases per 100,000 individuals [1–3]. In the general population, ABC has a predilection for children and young individuals, is diagnosed more commonly in the second decade of life and has a male to female ratio of 1:1.16 [3]. In a purely pediatric multicenter study of 156 children that included 100 boys and 56 girls by Cottalorda et al. [1], the median age of diagnosis of ABC was 9.4 years (range: 18 months to 16 years) with the majority of patients (76 children) being older than 10 years. In the same article, the authors conducted a literature review of ABC in 18 pediatric studies that compiled 411 patients, including 212 boys and 199 girls, and found a mean age of diagnosis of 10.2 years (range: 18 months to 17 years) and again most cases occurred in patients older than 10 years of age.

Patients with ABCs typically present with insidious onset of pain, swelling or a palpable mass. Some variability in clinical presentation exists because ABCs, even in the same location, can have different growth rates with doubling times ranging from months to years. This variability in growth rate also contributes to the differences in imaging appearance. Acute pain may occur in patients who present with a pathological fracture. When the lesion involves the skull or spine, focal neurological symptoms (e.g., paresthesias, numbness) can accompany the pain or scoliosis [4–7].

In the last WHO Classification of Tumors of Bone (2020), there has been a shift in nomenclature in which the terms “ABC” and “ABC-like changes” that are found within certain preexisting primary bone neoplasms are suggested instead of “primary ABC” and “secondary ABC,” respectively [8]. ABC is considered a primary bone lesion in 70% of cases, whereas the remaining 30% of cases represent ABC-like changes associated with different primary bone tumors [9]. Bone tumors that have been associated with aneurysmal changes reminiscent of ABC in the general population include benign tumors such as chondroblastoma, fibrous dysplasia, giant cell tumor, osteoblastoma, and non-ossifying fibroma and malignant tumors such as osteosarcoma [10, 11]. In the studies by Sasaki et al. [10] and Gutierrez et al. [11], the most common tumors associated with ABC-like changes were giant cell tumor and chondroblastoma, both of which typically involve the epiphysis. Other types of ABC include the solid variant and ABC of the soft tissues.

In this article, we first discuss the pathophysiology, histology and distribution of ABC and its variants. We then discuss the imaging appearance of ABC by focusing on radiographs, magnetic resonance imaging (MRI) and computed tomography (CT) as well as the differential diagnosis, specifically telangiectatic osteosarcoma, which is a true mimicker of ABC. Finally, we discuss the various therapeutic options for ABC, focusing on image-guided therapy, surveillance and clinical outcomes.

## Pathophysiology

Several theories have been proposed over the years regarding the pathophysiology of ABC. In 1950, Lichtenstein [12] proposed that ABC was a reactive lesion to regional vascular disturbances that led to increased intraosseous pressure, eventually causing bone destruction and expansion. Another theory proposed a traumatic etiology, followed by an aberrant reparative process [13]. The recent identification of recurrent chromosomal translocations involving the USP6 gene confirmed that ABC and its solid variant are clonal neoplastic processes [14–16]. A USP6 rearrangement is present in approximately 65% to 70% of ABC cases, with CDH11-USP6 fusion in 30%. Additional fusion partners have also been described in the literature [17–20].

USP6 is a ubiquitin-specific protease whose gene is localized to the short arm of chromosome 17(17p13.2). USP6 plays an important role in the regulation of several processes, such as protein stability and degradation, cell signaling, angiogenesis and inflammatory response. The oncogenic mechanism by which USP6-related gene fusion is involved in tumorigenesis (as in ABC) is beyond the scope of this article, but the interested reader is referred to references [14, 15]. It is important to note that rearrangements in the USP6 gene are not unique to ABC and that they have been described in other benign entities, such as nodular fasciitis, myositis ossificans, fibro-osseous pseudotumor of digits and in a subset of fibroma of tendon sheath, all sharing similar clinical and histopathological findings with ABC. Because of these similar histological and cytogenetic findings, some authors have proposed to call these lesions USP6 associated neoplasms (UAN) [18]. Due to these unique cytogenetic findings, fluorescent in situ hybridization (FISH) and next generation sequence (NGS) are useful tools in differentiating ABC from other primary bone lesions with ABC changes, which typically lack USP6 rearrangements [15, 21]. USP6 analysis is not universally available, has a prolonged processing time and is costly; therefore, it is not routinely used in the diagnosis of ABC.

Several classifications have been proposed based on natural history, activity and morphological appearance of ABC. In 1969, Dabska and Buraczewski [22] were the first to reference the natural history of ABC by dividing the progression into four phases: (1) the initial osteolytic phase in which the cortices of the bone are involved with subtle periosteal reaction; (2) the growth phase characterized by progressive bone destruction with poor demarcation of the borders and no septations seen on radiographs, (3) the stabilization phase that leads to the typical ABC appearance of a well-defined, expansile lesion with sclerotic borders and internal septations and (4) the healing phase characterized by progressive ossification of the lesion.

**Fig. 1:**
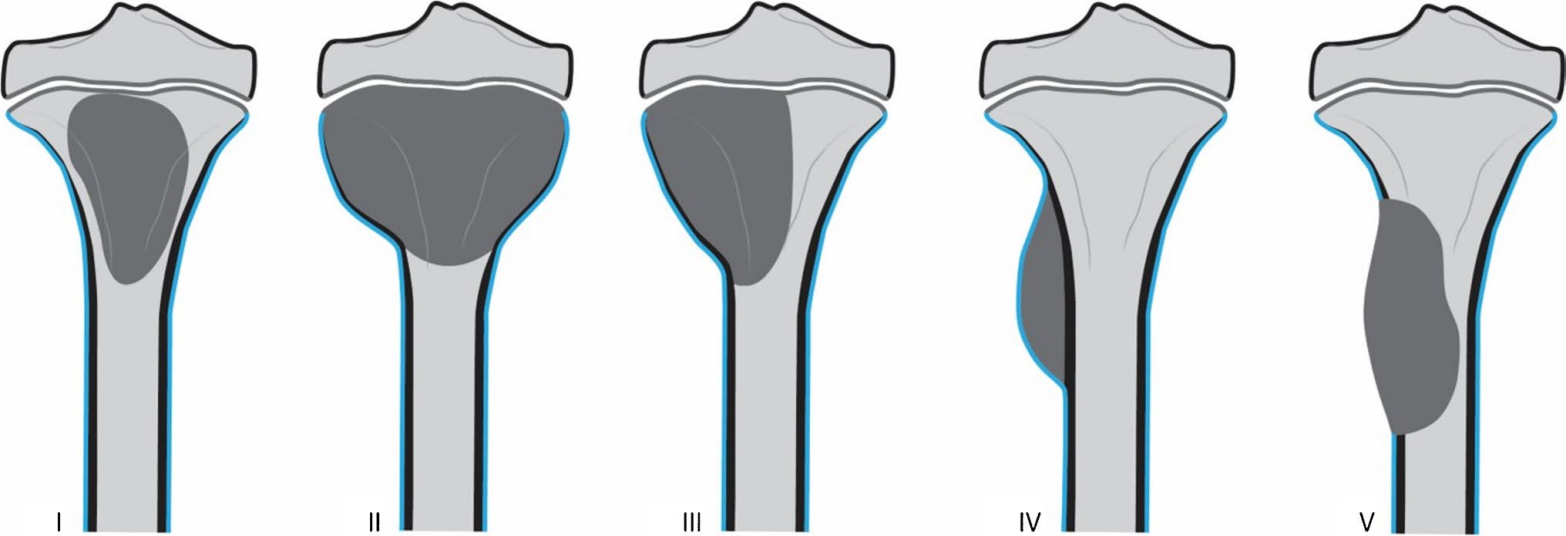
Aneurysmal bone cyst classification according to morphology. Reproduced with permission from Capanna et al. [23]. Type I: centrally located lesions that are well contained with no outline or slightly expanded outline. Type II: very expansile tumors with cortical thinning involving the entire bone segment. Type III: eccentric metaphyseal lesions that typically involve only one cortex. Type IV: subperiosteal lesions growing away from the bone. Type V: periosteal lesions expanding peripherally to ultimately penetrate the underlying cortex

**Fig. 2:**
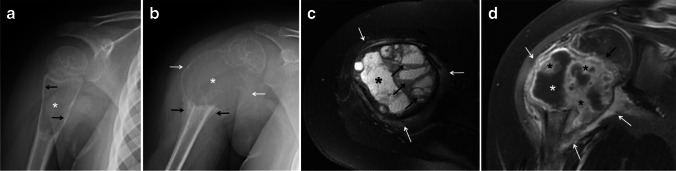
Rapid progression of a proximal humeral aneurysmal bone cyst from a type I to a type II in a 5-year-old girl. **a** An initial anteroposterior radiograph of the shoulder shows a proximal metadiaphyseal, lytic lesion (*asterisk*) of the humerus. The lesion causes endosteal scalloping (*arrows*), is slightly expansile and has no internal septations or matrix. **b** An anteroposterior radiograph of the shoulder obtained 7 months later shows significant interval growth of the lesion (*asterisk*) with blurring of the physeal margin. The lesion is now expansile and has marked cortical thinning (*white arrows*) with no sclerotic border or areas of mineralization. Aggressive periosteal reaction (*black arrows*) is identified at the distal margin of the lesion. **c** An axial fat-suppressed T2-weighted magnetic resonance (MR) image of the humeral lesion shows the expansile, multiseptated humeral lesion with a dominant cystic cavity (*asterisk*), fluid-fluid levels (*black arrows*) and mild adjacent soft-tissue edema (*white arrows*). **d** A coronal fat-suppressed, contrast-enhanced T1-weighted MR image of the humeral lesion clearly shows the focal bone expansion and physeal involvement (*black arrow*). The lesion is multicystic; the dominant cyst (*white asterisk*) displays peripheral contrast enhancement and areas of enhancing solid tissue (*black asterisks*). A rim of peripheral enhancing edema is present (*white arrows*)

**Fig. 3:**
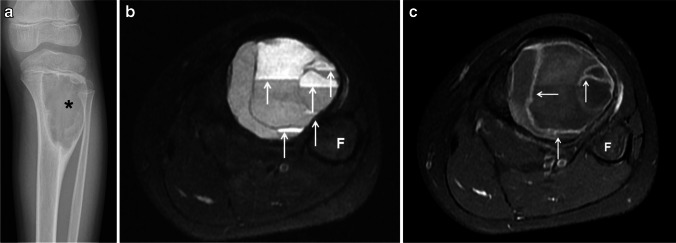
Type III aneurysmal bone cyst of the tibia in a 10-year-old girl. **a** An anteroposterior radiograph of the tibia shows an eccentric, well-defined, juxtaphyseal, lytic and expansile lesion (*asterisk*) involving the proximal tibial metadiaphysis. **b** An axial fat-suppressed T2-weighted magnetic resonance (MR) image of the leg shows fluid-fluid levels throughout the lesion (*arrows*). **c** A fat-suppressed, contrast-enhanced, T1-weighted MR image of the lesions shows thin, enhancing septations (*arrows*). *F* fibula

**Fig. 4:**
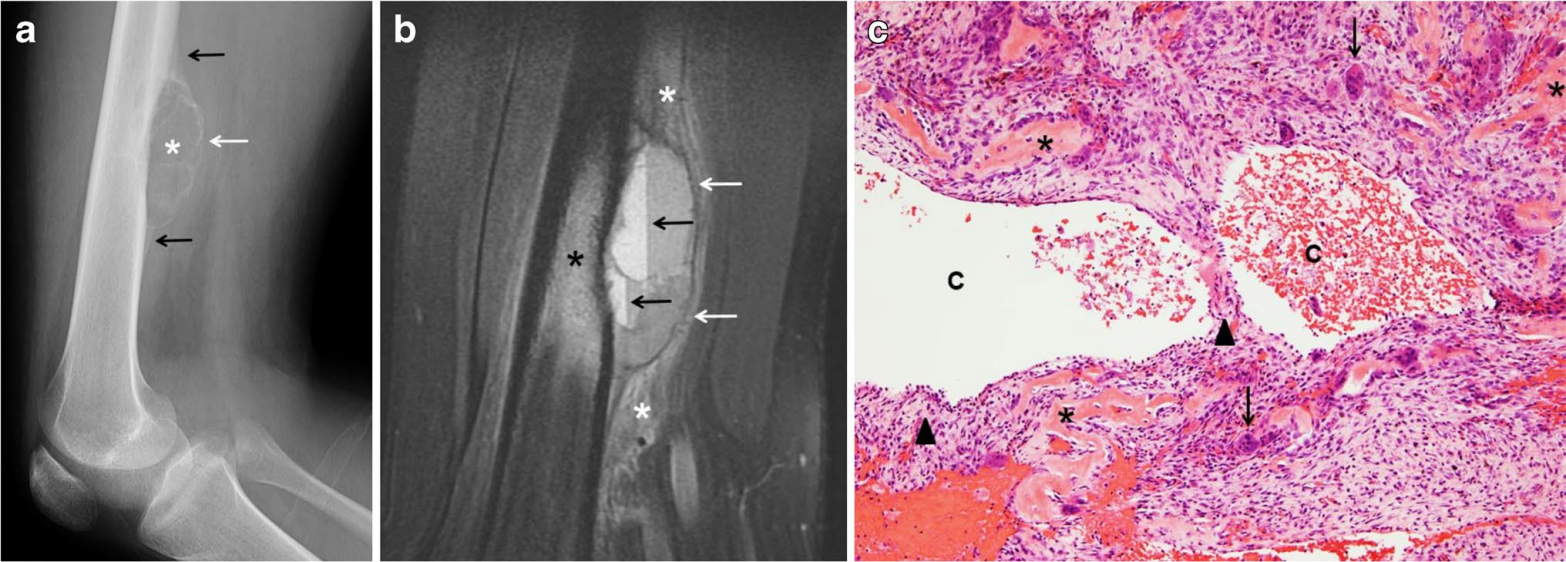
Type V aneurysmal bone cyst of the femur in a 16-year-old boy. **a** A lateral radiograph of the femur shows a superficial, parosteal, lytic and expansile lesion (*asterisk*) involving the distal femoral diaphysis. The lesion is exophytic extending into the adjacent soft tissues surrounded by a thin, calcified shell (*white arrow*). Periosteal reaction (*black arrows*) is identified at the proximal and distal margins of the lesion. **b** A sagittal fat-suppressed T2-weighted magnetic resonance image of the femur shows the exophytic mass involving the femoral cortex with a thin hypointense peripheral shell (*white arrows*) extending into the soft tissues. Fluid-fluid levels (*black arrows*) and adjacent bone marrow (*black asterisk*) as well as soft-tissue edema are present (*white asterisks*). **c** Hematoxylin and eosin staining, magnification 100X: Solid areas and cystic spaces (*C*) filled with blood, with cellular septa (*arrowheads*), benign osteoid (*asterisks*), stromal cells and giant cells (*arrows*)

In 1985, Capanna et al. [23] proposed a very useful classification according to the tumor morphology that could be applied to the radiographic and gross specimen appearance. This classification divided ABC into five morphological subgroups (Fig. 1). Type I represents centrally located lesions that are well contained with no outline or slightly expanded outline (Fig. 2). These lesions are more often found in the short bones of the hand and feet [24]. Type II comprises expansile tumors with cortical thinning involving the entire bone segment (Fig. 2). Type III represents the most common type, eccentric metaphyseal lesions, which typically involve only one cortex (Fig. 3). Type IV is the least common type of ABC. These lesions are subperiosteal and grow away from the bone. Type V corresponds to lesions based periosteally that expand peripherally and ultimately penetrate the underlying cortex (Fig. 4). In the same article, ABC was also classified based on the degree of activity in three distinct radiographic stages: (1) inactive when the tumor is contained and non-expansile, with internal septations, a sclerotic border and intact cortices; (2) active wherein the lesion becomes slightly symptomatic, as well as shows expansion, an indistinct border with cortical thinning and a distinct peripheral layer of reactive bone, and (3) aggressive when the tumor is most active and becomes rapidly expansile, causing destruction of the bone and extension into the surrounding tissues (Fig. 2) [23, 25, 26].

## Histology

ABC is currently classified as a benign osteoclastic giant cell-rich tumor on the 2020 WHO Classification of Tumors of Bone [8, 27]. Macroscopically, ABC is mainly composed of cystic spaces filled with blood and surrounded by a thin shell of reactive bone. Solid areas can also be present. Microscopically, ABC is characterized by solid areas and blood-filled cystic spaces that lack an epithelial or endothelial lining, separated by cellular septa. Overall, three main histological components are identified: (1) a cellular component that includes osteoclast-like multinucleated giant cells that express high levels of receptor activator of nuclear kappa B (RANK) and neoplastic stromal mononuclear and myo/fibroblastic cells that express high levels of RANK ligand (RANKL), (2) a fibrillar component that comprises collagenous extracellular matrix and (3) an osteoid component made up of organic bone matrix deposited by osteoblasts. Mitoses are usually conspicuous; however, cytologic atypia should not be present and necrosis is uncommon (Fig. 4). RANKL is a tumor necrosis factor that stimulates osteoclasts by binding to RANK. This mechanism is not unique to ABC as it can also occur in other tumors, such as giant cell tumor and chondroblastoma, which explains in part the lytic component seen in these lesions [27, 28].

The term “solid variant” ABC has been used interchangeably with a histologically indistinguishable entity, giant-cell reparative granuloma. The term “giant cell reparative granuloma” has commonly been used in the literature to describe lesions that involve gnathic sites, either the mandible or maxilla, or the short tubular bones of the hands and feet. On the most updated WHO Classification of Tumors of Bone (2020), this terminology has changed, as tumors located in the small bones of hands and feet likely represent the solid variant ABC [8]. Like ABC, these lesions were initially thought to be reactive and non-neoplastic; however, more recent studies confirmed a translocation involving the USP6 gene on chromosome 17p13 [16]. The terminology “giant cell reparative granuloma” is reserved for lesions in the gnathic location. The main histological distinction of solid variant ABC is the lack of blood-filled cystic spaces, hence a predominantly solid architecture [27].

ABC-like changes can be associated with several aforementioned primary bone lesions, with focal histological features similar to those seen with ABC; however, rearrangements of the USP6 gene at chromosome band 17p13.2 are not found [8]. Therefore, extensive sampling of the lesion, especially of any solid component if present, and occasionally genetic testing are sometimes required to distinguish ABC from other primary bone neoplasms containing ABC-like changes.

## Distribution

**Fig. 5:**
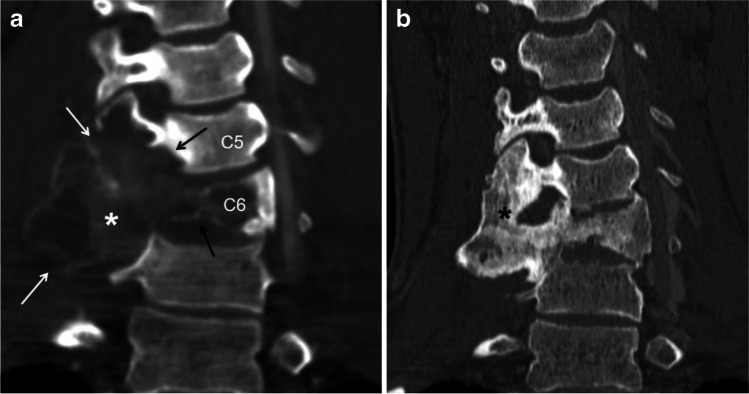
Aneurysmal bone cyst (ABC) of the cervical spine involving two consecutive vertebrae in a 16-year-old boy before and after percutaneous sclerotherapy with doxycycline. **a** A coronal reconstruction of a contrast-enhanced computed tomography (CT) scan image of the cervical spine using bone window shows a large, expansile lytic lesion (*asterisk*) involving the transverse processes (*white arrows*), articular pillars and body of C5 and C6 (*black arrows*). **b** A coronal reconstruction of a non-enhanced CT scan image of the cervical spine using bone window 6 years later after sclerotherapy shows complete interval healing of the lesion (*asterisk*) with diffuse sclerosis, remodeling of the bone and localized fusion at the site of the treated ABC

**Fig. 6:**
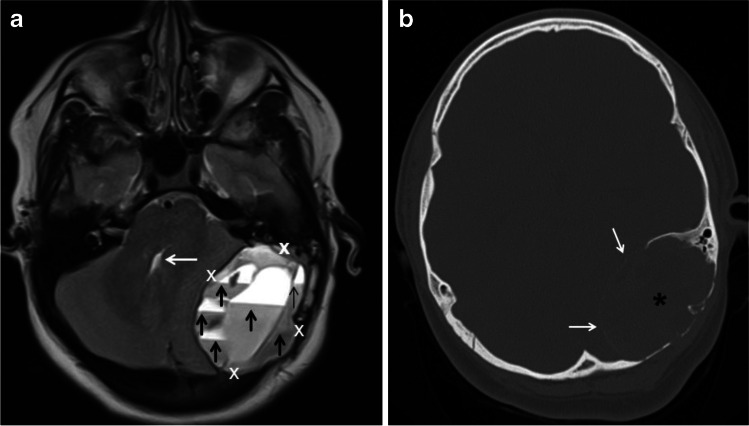
Aneurysmal bone cyst (ABC) of the skull and petrous bone in a 10-year-old boy. **a** An axial T2-weighted magnetic resonance image of the head shows a large, elliptical, expansile mass (calipers) in the left side of the posterior fossa causing mass effect upon the left cerebellar hemisphere and partial effacement of the fourth ventricle (*white arrow*). The mass is multiseptated and contains fluid-fluid levels of different signal intensity distributed throughout the lesion (*black arrows*). **b** An axial computed tomography scan image using bone windows shows to better advantage the petrous bone and occipital bone involvement by the ABC (*asterisk*) as well as a thin peripheral calcified shell (*white arrows*)

ABCs can involve every skeletal site, but the most common locations are the metaphysis of the long bones (Figs. 2 and 3), followed by the spine (Fig. 5). In a review of the pediatric literature by Cottalorda et al. [1], 62.7% of ABC occurred in the long bones with the femur (22.3%) and tibia (17.4%) being the most common sites followed by the spine (14.7%). Schreuder et al. [29] found a similar site distribution of ABCs in a review of the literature that included both children and adults. ABC can involve any part of the spine, including the sacrum. In the mobile spine, the most common location is the lumbar, followed by the cervical and then the thoracic spine [1, 30]. Although any part of a vertebra can be involved, ABC has a predilection for the posterior elements. In addition, ABC can involve consecutive vertebral bodies (Fig. 5) and result in vertebra plana [4, 6, 7, 31, 32]. ABC can also involve the flat bones of the pelvis, with a predilection for the acetabular triradiate cartilage area [4]. Pelvic ABC may become prodigious before diagnosis due to the deep location [1]. In the head, ABC can involve any bone of the skull and face, including the petrous bone (Fig. 6) and paranasal sinuses [33, 34].

## Imaging

### ABC

As with any other bone tumor, the first imaging modality to evaluate a patient with a suspected ABC is radiography. MRI or CT are helpful modalities to confirm the diagnosis, to further characterize the lesion, which may help in preprocedural planning, and to assess the risk of pathological fracture. In their study, Mahnken et al. [35] found that MRI had a similar sensitivity and slightly higher specificity compared to conventional radiographs in the diagnosis of ABC; however, the concomitant use of both modalities improved the sensitivity, specificity, positive predictive value and interobserver agreement.

**Fig. 7:**
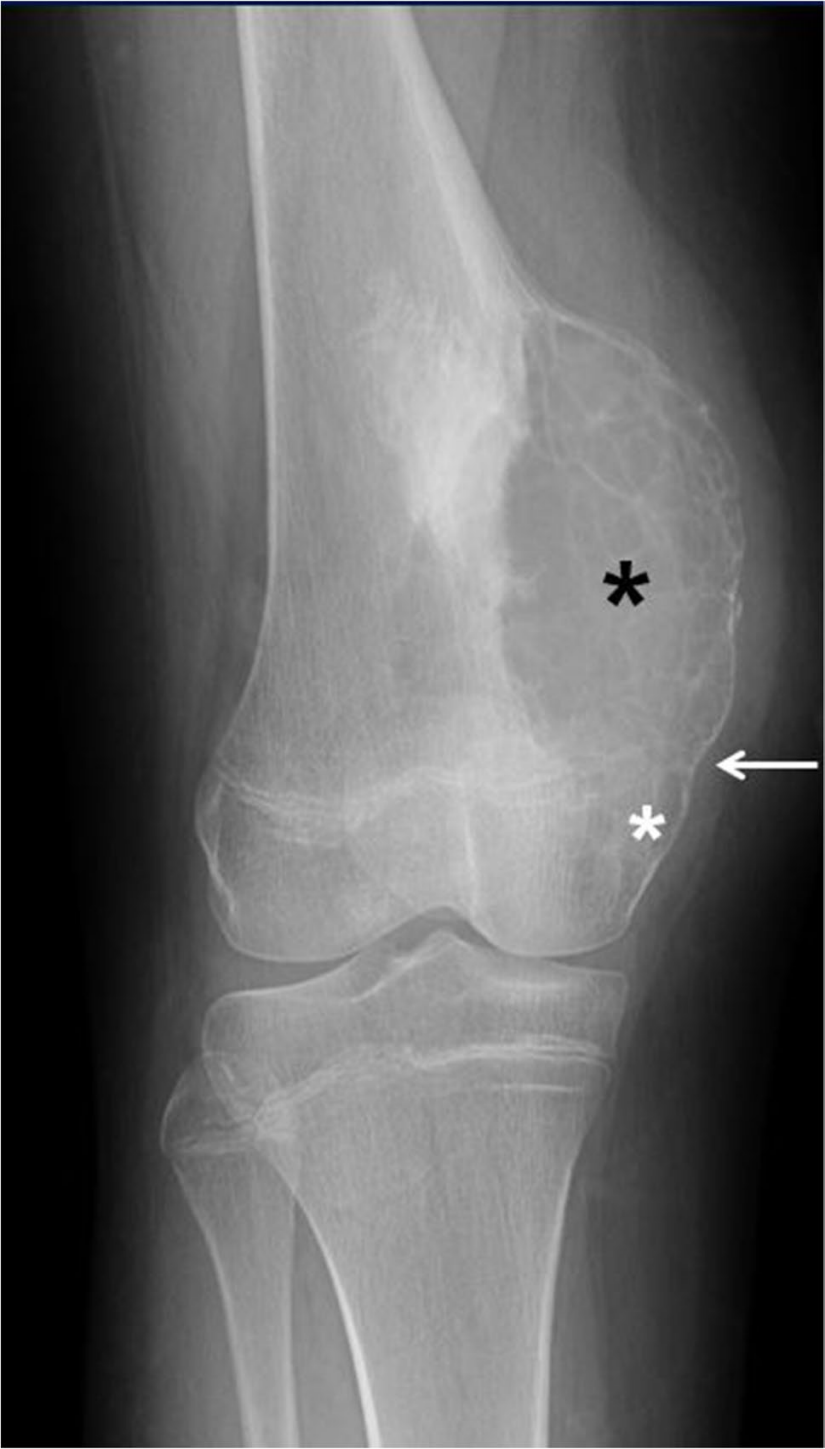
Epiphyseal extension of an aneurysmal bone cyst of the left distal femur in a 14-year-old girl. An anteroposterior radiograph of the left femur shows a well-defined, lytic, expansile metadiaphyseal lesion (*black asterisk*) with multiple internal septations causing cortical thinning. There is clear epiphyseal extension (*white asterisk*) of the lesion across the physis (*arrow*)

Typically, ABCs on radiographs are lytic, expansile, geographical, lobulated, metaphyseal-based lesions and have a distinct sclerotic border (Fig. 3). ABC also has internal septations that may (Fig. 7) or may not (Fig. 3) be visible on radiographs. Different types of periosteal reaction may occur [24, 35, 36]. In cases of an associated healing pathological fracture, nonaggressive periosteal reaction (e.g., smooth, lamellated) can be observed. On the other hand, during the most active phase, benign or aggressive periosteal reaction that can simulate Codman triangle (a radiographic feature more commonly associated with osteosarcoma) has been reported (Fig. 2) [37]. In a study of 997 bone tumors by Wenaden et al. [38], five ABCs in the active phase displayed periosteal reaction interpreted as Codman triangle. Most ABCs are metaphyseal and eccentric in location (Fig. 3); however, diaphyseal lesions (Fig. 4) may occur. Furthermore, as proposed by Capanna et al. [23], ABC can also be centrally located or have a periosteal or subperiosteal location within the involved bone. Aggressive forms of ABC can cause cortical disruption and extend into the overlying soft tissues (Fig. 2) and when juxtaphyseal, it can cross the physis into the epiphysis (Fig. 7), sometimes resulting in growth disturbance and deformity. When the lesion extends into the adjacent soft tissues, it is typically surrounded by a delicate, thin osseous rim described as a “shell,” which is an important feature suggestive of a benign process (Figs. 4 and 7) [4, 39–41].

MRI, besides lacking ionizing radiation, has the advantage of a better contrast resolution, thus allowing a precise delineation of the extent of the lesion (e.g., physeal/epiphyseal involvement, soft-tissue extension), perilesional edema and the internal composition (e.g., fluid-fluid levels, enhancing nodularity, septations, soft-tissue mass). Fluid-fluid levels of different signal intensity, which are better identified on MRI (especially on fluid sensitive sequences (Figs. 2 and 3) compared to CT scan, are highly suggestive but not pathognomonic of ABCs [37, 42]. In a study of 53 patients, Rajeswaran et al. [42] found that fluid-fluid levels distributed throughout a bone lesion **(**Fig. 6**)** was almost always indicative of benign process and 78% of the lesions corresponded to ABCs. Soft-tissue and bone marrow edema (Fig. 4), which are best depicted on MRI due to its high contrast resolution, can be present in ABCs. In the study that included 42 patients by Mahnken et al. [35], approximately one-third of ABCs exhibited perilesional edema, a sign that was attributed to rapid growth in more aggressive lesions and not necessarily related to malignancy. Peripheral and septal enhancement (Fig. 3) and even internal islands of enhancing, solid soft tissue (Fig. 2), can be observed following the administration of intravenous gadolinium [24, 36]. Mahnken et al. [35] found that 96.7% of ABC had some form of contrast enhancement on MRI. ABCs displayed mainly peripheral enhancement in 52.5%, septal enhancement in 35.8% and central diffuse enhancement in 11.7%. In the same study, the authors found that septal enhancement was much more common with ABC than with other bone lesions (8.3%) [35]. CT allows 3-D reconstruction that is useful in complex anatomical locations, such as the spine (Fig. 5), skull (Fig. 6), facial bones, pelvis or hindfoot, and may obviate the need of sedation due to its rapid acquisition. CT can also identify areas of mineralization, cortical destruction and periosteal reaction more readily than MRI.

### ABC-like changes associated to certain bone neoplasms

**Fig. 8:**
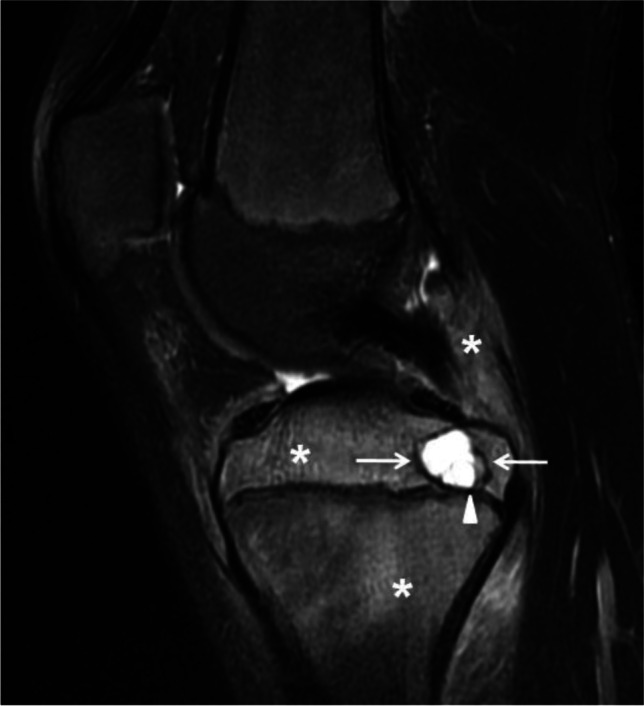
Chondroblastoma containing aneurysmal bone cyst (ABC)-like changes in a 13-year-old boy. A sagittal fat-suppressed T2-weighted magnetic resonance image of the knee shows a well-defined, lobulated lesion involving the posterior aspect of the proximal tibial epiphysis (*arrows*) corresponding to the chondroblastoma. The lesion is cystic and multiseptated containing a fluid-fluid level (*arrowhead*). Note the extensive, surrounding bone marrow and soft-tissue edema (*asterisks*) characteristic of chondroblastomas. After resection, the diagnosis of chondroblastoma with ABC-like changes was confirmed

ABC tends to occur in the first two decades of life, presenting with the typical radiographic features of an expansile, lytic, eccentric and metaphyseal lesion with a sclerotic border. On the other hand, the age of presentation, anatomical location and imaging features of primary bone tumors containing ABC-like changes are more variable and, aside from the presence of fluid-fluid levels, tend to reflect the features of the underlying lesions [10, 11]. Location is also an important clue in the diagnosis; for example, in cases of fibrous dysplasia and non-ossifying fibroma, the lesion is diaphyseal; in chondroblastoma, the lesion is epiphyseal with an extensive, surrounding inflammatory reaction (Fig. 8); in giant cell tumors, the lesion is predominantly epiphyseal but can be metaphyseal in skeletally immature patients [43]. An associated soft-tissue mass, unusual in ABC, is suggestive of a giant cell tumor or a malignancy, such as osteosarcoma, containing ABC-like changes [10, 11]. A pure epiphyseal location of a lesion with fluid-fluid levels should raise suspicion of neoplasm containing ABC-like changes rather than an ABC.

### Solid variant ABC

**Fig. 9:**
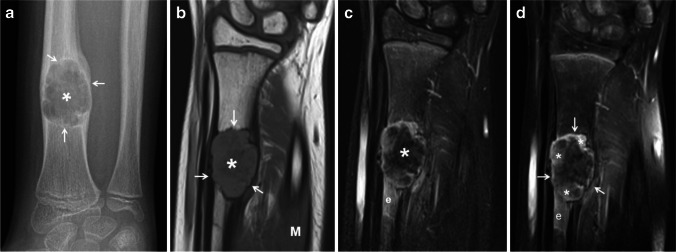
Solid variant aneurysmal bone cyst of the right radius in a 9-year-old girl. **a** An anteroposterior radiograph of the right wrist shows an expansile, lytic lesion (*asterisk*) involving the distal diaphysis of the radius. The lesion is geographical with endosteal scalloping and a sclerotic border (*arrows*). Minimal nonaggressive periosteal reaction is present proximally. **b** A coronal T1-weighted magnetic resonance (MR) image shows the lesion (*asterisk*) as solid and expansile with a slightly hyperintense parenchyma compared to muscle (*M*) and a sclerotic border (*arrows*). **c** A coronal fat-suppressed T2-weighted MR image shows the expansile lesion (*asterisk*) as heterogeneous in signal but predominantly hypointense. Note the adjacent bone marrow edema (*e*). **d** A coronal fat-suppressed, contrast-enhanced, T1-weighted MR image shows patchy, heterogeneous contrast enhancement (*asterisks*) by the lesion (*arrows*) and by the adjacent bone marrow edema (*e*)

Solid variant ABC exhibits more radiographic variability than the “classic” ABC. Similar to its classic counterpart, when involving the long tubular bones, solid variant ABC has a predilection for the lower extremities with the femur, followed by the tibia, as the most commonly affected sites. It is more frequently eccentric and metaphyseal or diaphyseal in location, and it can also involve any part of the bone, including the cortex and periosteum. Radiographically, solid variant ABC is typically lytic and expansile (Fig. 9) with a variable degree of mineralization; however, it can be non-expansile in approximately one-third of the cases [44]. On MRI, solid variant ABC is predominantly solid; however, cystic spaces containing fluid-fluid levels, but lacking internal septations, may be present in larger lesions. On T1-weighted, these lesions are iso to slightly hyperintense to muscle (Fig. 9) and heterogeneously hyperintense on fluid-sensitive sequences with patchy hypointensities that likely represent areas of mineralization (Fig. 9). Following the administration of gadolinium, solid variant ABC usually displays contrast enhancement (Fig. 9) that can be intense. Surrounding soft-tissue and bone marrow edema can be significant in solid variant ABC, especially small tumors [44, 45].

## Differential diagnosis

The radiographic appearance of ABC is varied radiographically as lesions can involve virtually every bone in the body and any part of a bone. The main differential diagnosis of ABC in the long bones includes unicameral bone cyst (UBC), giant cell tumor and telangiectatic osteosarcoma. Being a malignant bone tumor, telangiectatic osteosarcoma clearly poses the greatest diagnostic challenge; however, it is very rare in the pediatric population. Giant cell tumors typically involve the epiphysis and affect skeletally mature patients, with a peak incidence in the third and fourth decades. In skeletally immature patients, giant cell tumor is rare but may be found in the metaphysis [43]. Although it is true that USP6 is positive in 70% of ABC cases, but not in giant cell tumors, it is also true that a USP6 negative tumor is still much more likely to be an ABC than a giant cell tumor, which is a very rare occurrence in the pediatric population.

### Unicameral bone cyst (UBC)

Radiography is the main modality of choice in the evaluation of suspected bone tumors, and in the case of an UBC is usually diagnostic. UBC is most frequently diagnosed in the second decade of life. Patients are often asymptomatic but may present with pain due to a pathological fracture after minor trauma. UBC is a metaphyseal-based lesion present at the ends of long bones; most often, the proximal humerus, followed by the femur. These two locations account for more than 80% of UBC cases. [24, 46]. Diaphyseal locations may occur but are rare. Radiographically, UBC is typically seen as a geographic, lytic lesion with a sclerotic rim. UBC is centrally located within the medullary cavity following the longitudinal axis of the bone, exhibits smooth endosteal scalloping and generally no bone expansion. Periosteal reaction, internal matrix, septations and cortical disruption are not typical features unless there is an associated pathological fracture. In cases of a pathological fracture, the “fallen bone fragment,” considered by some as pathognomonic, may be observed. MRI is not routinely used to diagnose UBC; however, unlike ABC, no internal septations or fluid-fluid levels are present, except after a pathological fracture or previous intervention [24, 31, 47].

### Telangiectatic osteosarcoma

When an ABC is suspected, the main challenge is to differentiate it from telangiectatic osteosarcoma, which can mimic an ABC in all aspects: age and clinical presentation as well as histologically and radiographically. Patients with telangiectatic osteosarcoma present with localized pain and swelling/mass and sometimes a pathological fracture [48, 49]. Pathological fracture occurs more commonly in telangiectatic osteosarcoma (30%) compared to ABC (15%) due to relatively rapid tumor growth and extensive bone destruction [50]. Telangiectatic osteosarcoma has no gender predilection, similar to ABC. Likewise, the age of presentation is also generally the second decade [37, 49, 50].

**Fig. 10:**
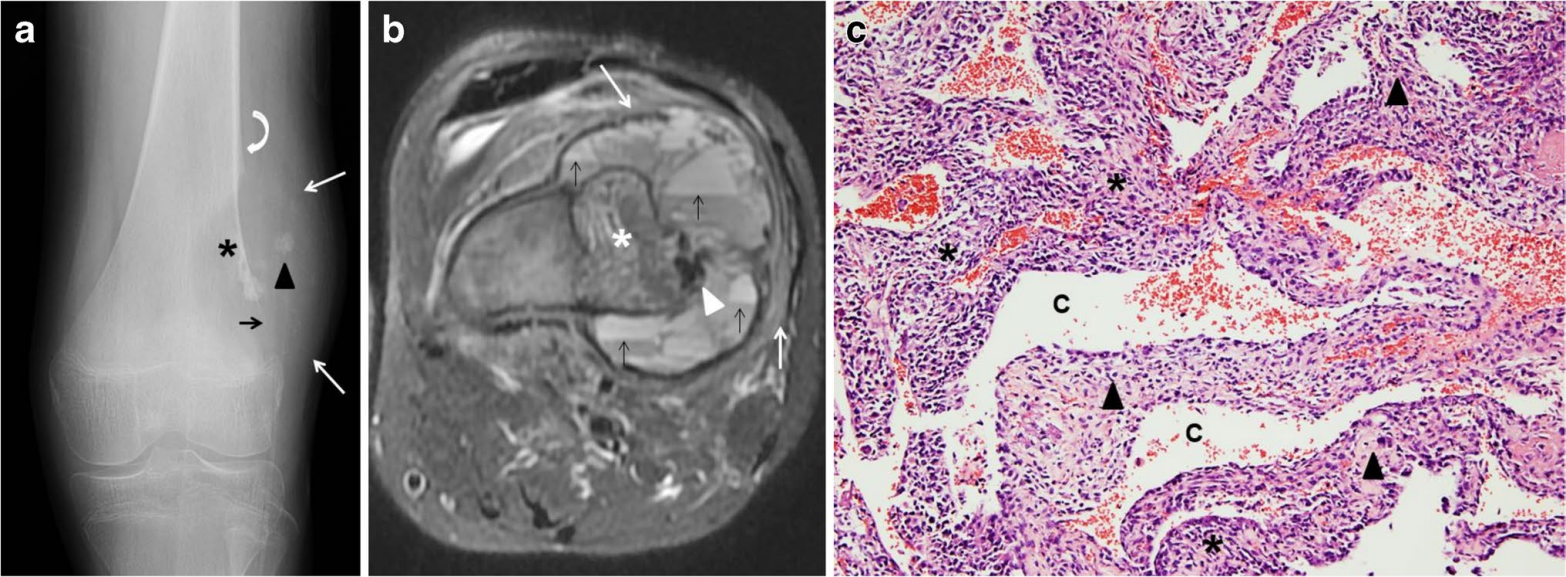
Telangiectatic osteosarcoma in a 17-year-old boy. **a** An initial anteroposterior radiograph of the knee shows a distal femoral metadiaphyseal, geographical, eccentric, lytic lesion (*asterisk*) with no sclerotic border. There is focal destruction of the distal femoral cortex (*black arrow*), focal areas of mineralization (*arrowhead*) and an associated soft-tissue mass (*white arrows*). Codman’s triangle is present proximally (*curved arrow*). **b** An axial fat-suppressed T2-weighted magnetic resonance image of the femoral lesion shows the eccentric, osseous mass (*asterisk*) with multiple fluid-fluid levels located in the periphery (*black arrows*), hypointense areas of mineralization (*arrowhead*) and adjacent soft tissue edema (*white arrows*). **c** Hematoxylin and eosin staining, magnification 100X: solid areas and cystic spaces (*C*) filled with blood, fibrous cellular septa (*arrowheads*), malignant stromal cells (*asterisks*)

Histologically, telangiectatic osteosarcoma is characterized by predominantly cystic, blood-filled spaces with fibrous septa similar to ABC; however, the septa in telangiectatic osteosarcoma contain atypical malignant stromal cells with nuclear hyperchromasia, marked pleomorphism and atypical mitosis. A lace-like neoplastic osteoid may be present (Fig. 10). In addition to the cystic spaces, less atypical areas with benign multinucleated cells admixed with stromal cells, and hemosiderin-laden macrophages, can be seen. Telangiectatic osteosarcoma also exhibits cytogenetic abnormalities associated with osteosarcoma, such as mutations in TP53 and retinoblastoma (RB) genes among others, and it lacks the USP6 gene rearrangement seen in ABC, which can be helpful in supporting the diagnosis [48].

**Fig. 11:**
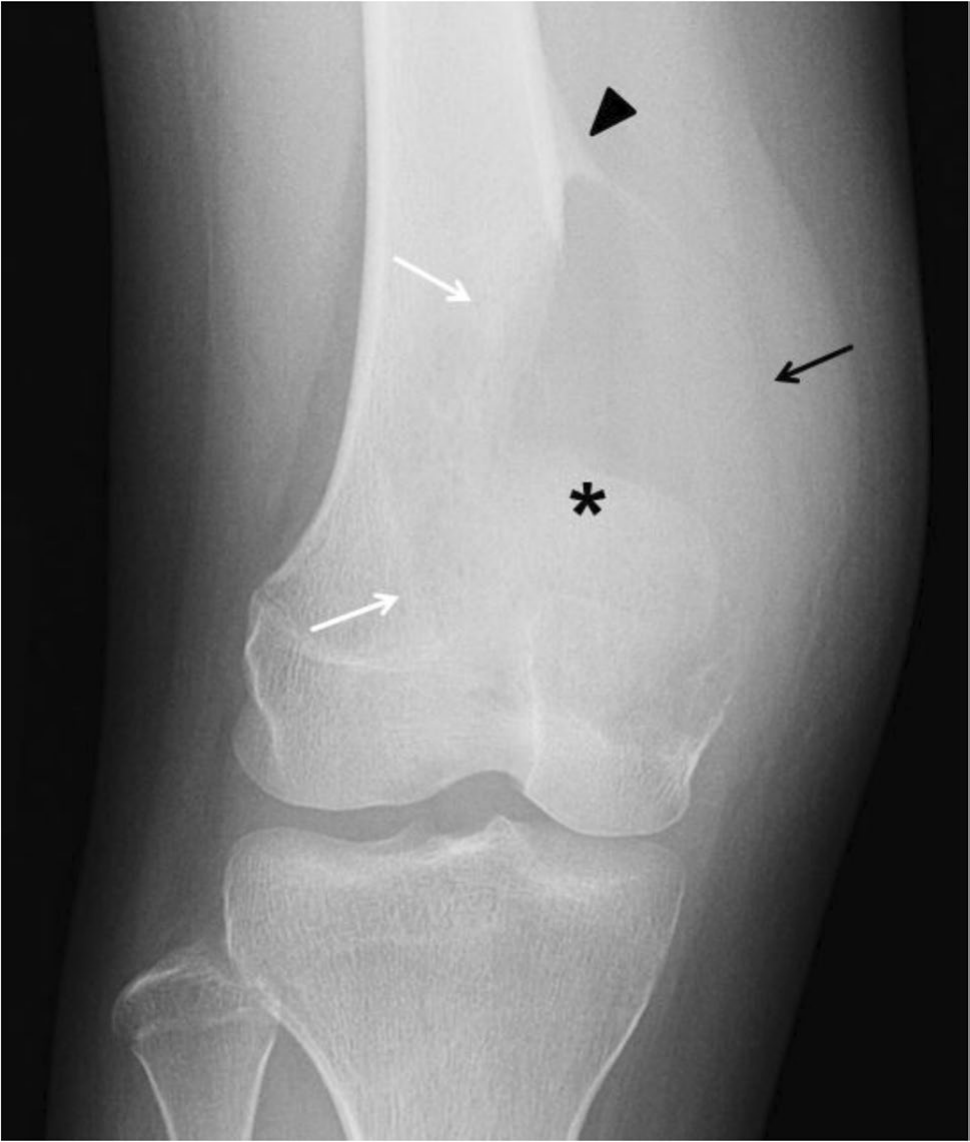
Radiographically aggressive, biopsy proven aneurysmal bone cyst (ABC) of the distal femur in a 17-year-old boy (compare the striking similarity with patient from Fig. 10). An anteroposterior radiograph of the distal left femur shows a lytic distal metadiaphyseal lesion (*asterisk*) of the left femur. The lesion extends into the epiphysis and adjacent soft tissues with no calcified peripheral shell (*black arrow*). The ABC has a wide zone of transition (*white arrows*) and aggressive periosteal reaction proximally (*arrowhead*). The lesion was biopsied twice and ABC was confirmed twice

The imaging features of telangiectatic osteosarcoma can also be strikingly similar to those of ABC (Fig. 11); therefore, attention to subtle clues is important to suggest the diagnosis. Telangiectatic osteosarcoma on radiographs appears as an expansile, lytic lesion, with a wide zone of transition, cortical disruption and an associated soft-tissue mass that may contain areas of mineralization. Telangiectatic osteosarcoma typically causes geographical bone lysis lacking the sclerotic margin typically seen with ABC, which is a very important differentiating feature (Fig. 10). However, an ABC during the growth phase may also lack a distinct border and grow rapidly (Fig. 2). Telangiectatic osteosarcoma, like ABC, has a metaphyseal predilection of the long bones, more frequently, the femur; but unlike ABC, other locations such as the axial skeleton are uncommon [37]. Imaging features of telangiectatic osteosarcoma on cross-sectional imaging (e.g., CT and MRI) include fluid-fluid levels, internal solid component, septa and areas of mineralization. Telangiectatic osteosarcoma, being a rapidly growing and expansile lesion, causes internal hemorrhage that frequently leads to the formation of fluid-fluid levels (Fig. 10), a characteristic feature of ABC [51]. The distribution of fluid-fluid levels in telangiectatic osteosarcoma is another important differentiating feature from an ABC, as they tend to be incomplete and regional in telangiectatic osteosarcoma [37, 42]. Careful evaluation of the septa and the presence of a mineralized matrix are also important in making the correct diagnosis. Septa in telangiectatic osteosarcoma, when present, tend to be thick and display nodular enhancement as they correspond to the viable tumoral cells. Mineralized osteoid matrix, typically not seen in ABC, is often subtle in cases of telangiectatic osteosarcoma [49, 52]. Mineralized matrix is better identified on a CT scan (85%) than on radiographs (58%) and can be easily overlooked on MRI [52]. In the retrospective study by Zishan et al. [37] that included 183 pediatric and adult patients, the following features favoring ABC over telangiectatic osteosarcoma were suggested: smaller tumor size (maximum mean dimension 46 mm compared to 95 mm for telangiectatic osteosarcoma), the absence of soft-tissue mass, more than 2/3 of the lesion filled with fluid-fluid levels and thin septal enhancement following intravenous contrast administration [37].

## Treatment

Although the radiographic and histological features of both ABC and telangiectatic osteosarcoma have been described, the differentiation between them is still challenging for radiologists and pathologists. Because it can be difficult to differentiate an ABC from a malignancy based on imaging alone (Figs. 10 and 11), confirming the diagnosis by means of a biopsy before treatment is imperative [53–55]. Performing an image-guided core biopsy of a suspected ABC should be carefully planned with the orthopedic surgeon to discuss the most appropriate approach that would not compromise limb salvage. Ideally, the imaging and biopsy results should be discussed in a multidisciplinary team as it has been proven that a comprehensive approach (i.e. clinical, pathological and radiologic) improves diagnostic accuracy and optimal treatment for patients with bone tumors [56, 57].

In both ABC and telangiectatic osteosarcoma, viable (atypical, stromal) cells are predominantly localized within the septa and/or at the periphery of the predominantly cystic lesion (Figs. 3 and 10). This increases the difficulty in obtaining an adequate diagnostic sample when a large bore needle core biopsy or an open biopsy is performed. Fine needle aspiration biopsy can be an alternative as a solid specimen frequently cannot be obtained; however, interpretation requires significant expertise from a cytopathologist. Fine-needle aspiration biopsy may be able to distinguish a neoplasm from normal tissue, malignant from benign cells and even ascertain the grade of the tumor.

Many therapeutic alternatives have been performed over the years in patients with ABCs, from aggressive interventions, such as amputation and radiation therapy, to observation. Spontaneous healing of ABC has also been reported [58, 59]. The real incidence of spontaneous healing is difficult to calculate because most ABCs are treated after diagnosis. Spontaneous healing has also been reported after biopsy, possibly due to alterations in the existing equilibrium within the blood-filled cystic spaces. A conservative approach to ABC, with close radiographic follow-up, can be considered in select cases, especially in patients with small lesions and in sites that are at very low risk for fractures [59].

**Fig. 12:**
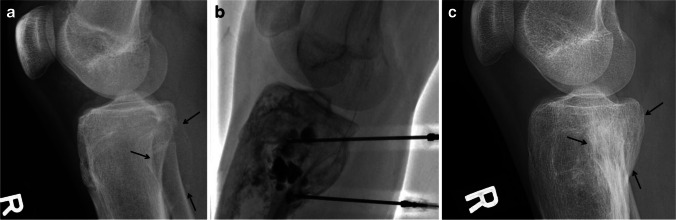
Type III aneurysmal bone cyst in a 16-year-old boy before, during and after percutaneous sclerotherapy with doxycycline. **a** A lateral radiograph of the proximal tibia shows an eccentric, well-defined, lytic and expansile lesion (*arrows*) involving the proximal tibial metaphysis with a faint peripheral calcified shell. **b** A fluoroscopic image of the lesion during sclerotherapy shows the insertion of two spinal needles and injection of contrast accumulating in several intralesional locules. **c** A lateral radiograph of the proximal tibia 6 years later after several treatments with sclerotherapy shows complete interval healing of the lesion (*arrows*) with some sclerosis and remodeling of the bone but no significant residual deformity

The goal of treatment in ABC is to promote healing of the cystic cavities to control pain, decrease the risk of pathological fracture and minimize deformity. The traditional treatment of ABC has been intralesional curettage with or without bone grafting, which is sometimes enhanced with adjuvant techniques, such as cauterization, phenol, argon bean coagulation and cryotherapy. Despite these techniques, local disease recurrence is relatively high. Wide local excision is rarely considered when there is extensive periarticular bone destruction or tumors in expendable bones like the clavicle or fibula. Over the past three decades, less invasive treatments using image-guided sclerotherapy with intralesional injection of various substances, such alcohol, polidocanol, doxycycline (Fig. 12) and calcitonin mixed with steroids, among others, have become an alternative to surgery. Sclerotherapy is a minimally invasive procedure; however, it may require multiple repeat injections [54, 60, 61]. In a critical systematic review by Bavan et al. [60], that assessed treatment efficacy for ABC involving the spine and long bones (28 articles that included children and adults), no controlled studies were found supporting any particular intervention. In this review, only one single-center, prospective study by Varshney et al. [55] that compared sclerotherapy using polidocanol with surgical treatment (intralesional excision with bone grafting) was found. This study, which included children and adults, used non-stratified randomization and lacked assessor blinding. The study found no statistically significant differences in the treatment success when comparing the injection and surgical treatment groups (93.3% vs. 84%, respectively). However, poorer functional outcomes and higher clinically important complications were seen with surgery [55, 60]. In a more recent, single-center, retrospective study of 74 pediatric and adult patients, which included ABC in the axial and appendicular skeleton, Deventer et al. [62] compared the most common techniques used in the treatment of ABC: intralesional curettage (34 patients), image-guided percutaneous polidocanol sclerotherapy (32 patients) and en bloc resection (8 patients). The authors found a local recurrence/persistent disease rate requiring further treatment in 44.1% of the group treated by intralesional excision. In the sclerotherapy group, persistent disease was seen in 90.6% of cases. Even though a complete cure was achieved in only 9.4% of cases with serial sclerotherapy, adequate healing with reduction in lesion volume was achieved in 71.9% of cases. Because the number of patients included in the en bloc resection was significantly small, the results were not comparable. This latter group also included larger lesions and lesions in more challenging locations, such as the spine, with a higher rate of complications. The results of the study [62] were similar to the study by Varshney et al. [55], suggesting that percutaneous sclerotherapy and intralesional curettage were at least equally efficient in the treatment of ABC. Both Bavan et al. [60] and Deventer et al. [62] concluded that prospective, multicenter, randomized control studies with standardized protocols on interventions for the treatment of ABC were needed to draw a solid conclusion. There are multiple studies advocating for sclerotherapy of ABC in spinal locations using various sclerosing agents [63–66]. Sclerotherapy of ABC in the spine is also routinely performed by the authors (Fig. 5). For more detailed information about ABC sclerotherapy technique, please refer to reference [59].

Selective arterial embolization has been predominantly used as an adjunct to surgery or sclerotherapy of ABCs in select cases [67, 68]. Image-guided, percutaneous thermal ablation has also been used in the treatment of ABC as a minimally invasive procedure [69]. Denosumab, a monoclonal antibody, has recently been used to treat ABC in an injectable form with promising results also in select cases and difficult locations based on the fact that ABCs belong to the osteoclastic, giant cell-rich tumors. There is evidence that giant cells in ABC have increased receptor-activator levels of nuclear kappa B (RANK) and increased expression of its ligand (RANKL). The RANK-RANKL signaling pathway promotes osteoclast activation; therefore, bone resorption and remodeling are directly inhibited by Denosumab [69, 70].

After treatment with curettage or sclerotherapy, good response is assessed radiographically by the decrease in ABC size, increased sclerosis with resolution of or reduction in the lytic component, increased cortical thickening and remodeling of areas of bone expansion (Figs. 5 and 12). Signs of response, especially after sclerotherapy, may take 2 to 3 months to be apparent on radiographs [61]. As serial sclerotherapy is often needed, healing will progress with each treatment for months to years following the last treatment. ABC recurrence, which usually occurs within 24 months following the final treatment, is diagnosed clinically with the return of symptoms (e.g., pain, pathological fracture). Signs of recurrence after treatment on follow-up imaging include interval enlargement of the mass or growing focal lucent areas within the lesion on radiographs and cystic areas with fluid-fluid levels on MRI. After sclerotherapy, localized non-ossified areas can remain inside the lesion that represent fibrofatty tissue rather that residual neoplasm, but they do not grow or cause symptoms [62, 71]. Several risk factors have been linked to tumor recurrence, including young age of the patient at diagnosis, particularly children younger than 5 years of age, male gender, and axial skeleton and juxtaphyseal locations of the lesion, which are technically difficult to remove due to fear of injury to the adjacent physis [71, 72]. Histologically, a mitotic index of 7 or higher has been linked to recurrence [73]. More recently, Docquier et al. [28] correlated the histology of the biopsy specimen with the risk of recurrence. The authors found that ABCs with increased cellularity containing predominantly stromal and giant cells had a higher risk of recurrence compared to those containing a predominant osteoid component. The lower risk of recurrence with the latter was linked to a healing phase in the natural history of the ABC.

## Conclusion

An ABC is a benign but aggressive lesion that affects predominantly children and young adults. On the most recent WHO Classification of Tumors of Bone (2020), ABC is considered a benign osteoclastic giant cell-rich neoplasm. ABC can affect any bone and any part of a bone, but the most common location is the metaphysis of a long bone. The imaging work-up of a suspected ABC starts with orthogonal radiographs. Cross-sectional imaging, especially MRI, should be considered complementary in order to confirm the diagnosis and to exclude other bone neoplasms. The imaging appearance of ABC widely varies according to the location and the stage of the tumor. The typical radiographic appearance of aneurysmal bone cyst is that of an eccentric, lytic, geographical, metaphyseal lesion with a sclerotic border with or without septations. On MRI, fluid-fluid levels are the hallmark of the lesion but are not pathognomonic as these are seen in several bone neoplasms. Telangiectatic osteosarcoma can have a very similar imaging appearance to ABC, especially in the presence of fluid-fluid levels; therefore, a biopsy is strongly suggested before treatment can be contemplated. Even then, the precise diagnosis can be difficult. Although both surgery and sclerotherapy are widely implemented for the treatment of ABC, there is no adequate evidence to support a particular therapeutic option.
